# (Pseudo)amide-linked oligosaccharide mimetics: molecular recognition and supramolecular properties

**DOI:** 10.3762/bjoc.6.20

**Published:** 2010-02-22

**Authors:** José L Jiménez Blanco, Fernando Ortega-Caballero, Carmen Ortiz Mellet, José M García Fernández

**Affiliations:** 1 Department of Organic Chemistry, Faculty of Chemistry, University of Seville, Prof. García González 1, Seville 41012, Spain; 2 Institute of Chemistry Research, CSIC-University of Seville, Americo Vespucio 49, Seville 41092, Spain

**Keywords:** carbopeptoids, glycoclusters, glycomimetics, pseudooligosaccharides, spaced sugars

## Abstract

Oligosaccharides are currently recognised as having functions that influence the entire spectrum of cell activities. However, a distinct disadvantage of naturally occurring oligosaccharides is their metabolic instability in biological systems. Therefore, much effort has been spent in the past two decades on the development of feasible routes to carbohydrate mimetics which can compete with their *O*-glycosidic counterparts in cell surface adhesion, inhibit carbohydrate processing enzymes, and interfere in the biosynthesis of specific cell surface carbohydrates. Such oligosaccharide mimetics are potential therapeutic agents against HIV and other infections, against cancer, diabetes and other metabolic diseases. An efficient strategy to access this type of compounds is the replacement of the glycosidic linkage by amide or pseudoamide functions such as thiourea, urea and guanidine. In this review we summarise the advances over the last decade in the synthesis of oligosaccharide mimetics that possess amide and pseudoamide linkages, as well as studies focussing on their supramolecular and recognition properties.

## Review

**Figure 1:**
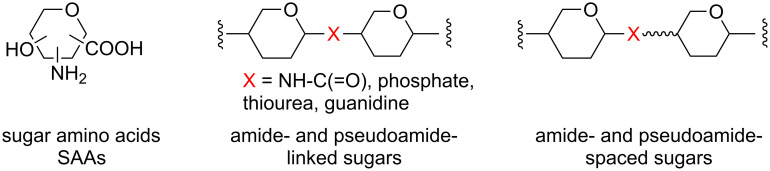
Schematic representation of sugar aminoacids (SAAs) and (pseudo)amide oligosaccharide mimetics.

Among the major classes of biomolecules, carbohydrates are characterized by nearly unlimited structural diversity. Monosaccharide units can combine to produce oligosaccharides in a number of permutations that increases rapidly with the number of units present, more so than is the case with other biomolecules such as polypeptides or oligonucleotides. This is determined by the stereochemical identity of the monosaccharide units present (e.g. glucose, galactose, mannose), their glycosidic linkage positions (e.g. 1→4, 1→6), the nature at anomeric centres (α or β), the presence of additional substituents such as sulfate or acyl groups and the overall degree of branching. The molecular diversity of oligosaccharide offers a valuable tool for drug discovery in the areas of biologically important oligosaccharides, glycoconjugates and molecular scaffolds by investigating their structural and functional impact. Currently, oligosaccharides are known to have functions in a broad variety of cell–cell interactions related to invading bacteria, viruses and cancer cells [1–4] and to play central roles in post-translational modifications of proteins [5–7], cell–cell communication [8] and immune response to pathogens [7,9–11]. However, the application of oligosaccharides as potential therapeutic agents has its main drawback in the low instability of natural carbohydrates in biological systems. In addition, solid phase synthesis of oligosaccharides is not yet efficient enough for generating oligosaccharide-based libraries that may be useful in the future for the discovery of new therapeutic drugs. Taking into account the increasing importance of glycobiology and the difficulties associated to the synthesis of carbohydrate-based libraries, several approaches based on the assembly of sugar building blocks through amide and pseudoamide linkages have been developed by different research groups over the last few years. Sugar aminoacids (SAAs, Figure 1) have extensively been used in the development of a large variety of molecules. In the past 10 years, some reviews on SAAs have summarised the different synthetic methods used to obtain such molecules [12–14] as well as their applications to access to a diversity of linear, branched and cyclic pseudooligosaccharides and glycomimetics often referred to as carbopeptoids [13–17]. In addition, SAAs have been widely used in the field of material sciences as carbohydrate-derived monomers for the design of novel chiral polyamides [18]. Nevertheless, these review articles mainly focus on amide-linked sugars and do not pay too much attention to other types of pseudosugars. A notable exception is the contribution by Wessel and Dias Lucas, who recently published an interesting review where oligosaccharide mimetics which deviate from the natural linking pattern were discussed [19]. This review also included pseudoamide-linked sugars (Figure 1).

In this current review we have tried to highlight the recent advances in the synthesis of oligosaccharide mimetics. We have focussed on amide and pseudoamide-linked oligosaccharides, and their molecular recognition properties. We intend this survey to be wide-ranging and cover the most recent trends in the field.

### Amide-linked sugars: carbopeptoids

The replacement of the glycosidic oxygen atom by an amide group leads to amide-linked sugars (Figure 1), which are synthesised by the sequential coupling of SAAs to form a class of compounds commonly referred to as carbopeptoids. The use of the amide functional group to connect sugar building blocks is inspired by the structure of peptides and the potential of carbohydrates to reproduce the structural features and biological properties of these polymers [12–13,17]. Poor bioavailability and metabolic stability of peptides have resulted in significant limitations as drug candidates. Another general problem is the loss of the original conformation in the isolated peptide fragments present in natural proteins, where the rest of molecule fixes a specific spatial disposition. Furthermore, short linear peptides cannot be restricted to adopt a particular conformation that enables effective interaction with a receptor [20]. As a consequence, a wide variety of methods to restrict the conformational freedom has been developed. One approach to get round this problem is the isosteric replacement of the amide bond in the peptide with a suitable mimetic to induce a specific secondary structure. Recent investigations have demonstrated that incorporation of SAAs in peptide structures can circumvent the adverse properties of bioactive natural systems. Thus, SAAs containing amino and carboxylic functional groups, and a rigid ring system (pyran, furan, oxetane) have emerged as versatile and conformationally constrained building blocks for the formation of peptide and oligosaccharide mimetics using standard peptide coupling techniques in solution or in the solid phase. Exploitation of the rigidity and diversity of the sugar backbone should permit subtle modifications and the rational design of oligomeric derivatives with tendency to adopt specific compact conformations (foldamers).

### Linear naturally-occurring SAA homo-oligomers

**Figure 2:**
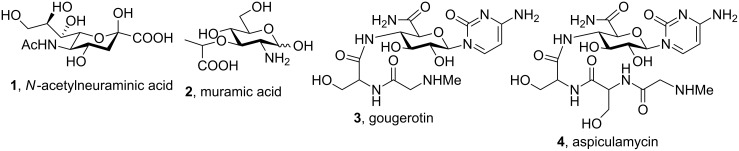
Natural SAAs structures and natural nucleosidic antibiotics.

SAAs occur extensively in nature as subunits of oligosaccharides in cell walls such as *N*-acetylneuraminic **1** and muramic acids **2**, as well as in some nucleoside antibiotics (e.g., gougerotin **3** and aspiculamycin **4**) (Figure 2).

**Scheme 1:**
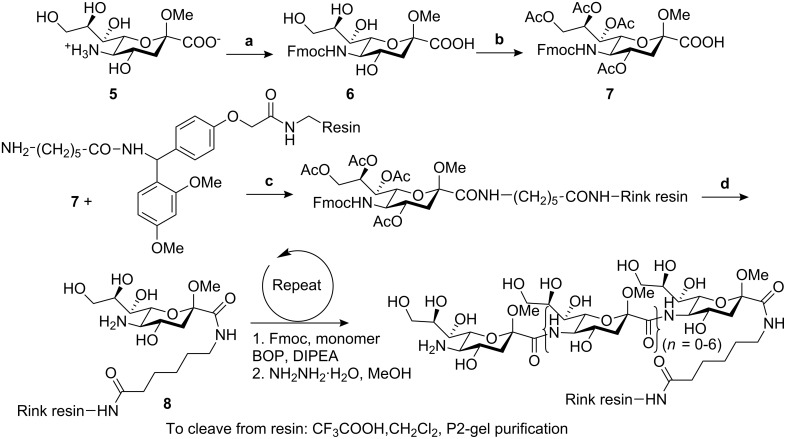
Synthetic route to the target amide-linked sialooligomers. (a) Fmoc-Cl, NaHCO_3_, H_2_O, dioxane, 0 °C. (b) Ac_2_O, Pyridine, 0 °C. (c) BOP, DIPEA. (d) NH_2_NH_2_·H_2_O, MeOH.

*N*-Acetylneuraminic acid is a readily available naturally occurring δ amino acid. By applying solid phase peptide methods on Rink resin with Fmoc protecting group chemistry [21–22], Gervay and co-workers have reported a series of (1→5) amide-linked sialooligomers ranging from dimeric to octameric species (Scheme 1) [23–24]. All of these were soluble in water, DMSO and methanol, which allowed NH/ND exchange NMR experiments and circular dichroism (CD) studies to be carried out. Combined structural studies showed that a tetramer is required for an ordered secondary structure [23,25].

### Linear non-naturally-occurring SAA homo-oligomers

**Figure 3:**
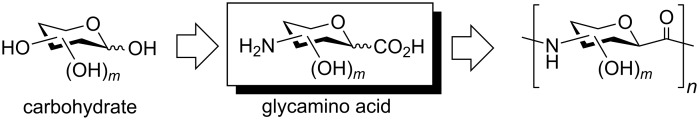
The general structure of glycoamino acids and their corresponding oligomers.

The first examples of amide-linked SAA-pyranose oligomers were reported as long ago as 1975 by Fuchs and Lehman [26–27]. In 2002, Ichikawa and co-workers developed a synthetic methodology to access oligomers of glycoamino acids, a family of non-natural SAAs with a carboxyl group at C-1 position and an amino group replacing one of the hydroxyl groups at either the C-2, 3, 4 or 6 positions (Figure 3).

**Figure 4:**
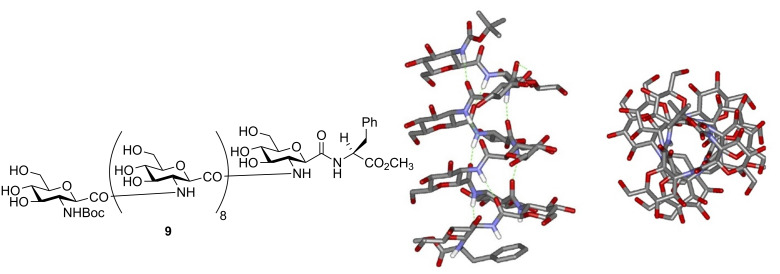
Conformational analysis of the β(1→2)-amide-linked glucooligomer **9**.

Glucose-type building blocks were prepared and used to construct β(1→2), β(1→3), β(1→4) and β(1→6)-linked homo-oligomers [28–30] that were found to form rigid secondary structures predetermined by the linkage position as evidenced from CD and NMR measurements. Moreover, conformational analysis by molecular modelling calculation on the β(1→2)-linked decamer **9** supported a helical arrangement, stabilised by a intramolecular hydrogen bonding in the form of a 16-membered ring, characteristic for β-peptides (Figure 4) [29]. Analogously, NMR and IR data of a β(1→6)-linked unsaturated glycamino acid tetramer showed a turn-like structure in chloroform solution [31].

Glycoamino acid-based carbopeptoids are resistant to glycosidases and exhibit interesting biological activities. Thus, the *O*-sulfated β(1→2) and β(1→6)-linked homo-oligomers effectively inhibit L-selectin-mediated cell adhesion, HIV infection and heparanase acitivity in a linkage-specific manner [28].

**Figure 5:**
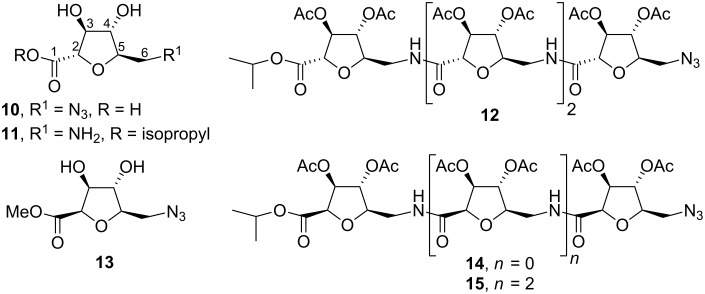
Short oligomeric chains of *C*-glycosyl D-*arabino* THF amino acid oligomers.

SAAs can have a wide range of structural diversity as a consequence of differing on ring sizes, structural manipulation (such as chain branching, deoxygenation, hydroxyl protection, unsaturation), relative position between amine and carboxylic function, and, perhaps most importantly, the spatial arrangement of the amine and acid moieties. The limitations of stereo-control of glycosidic linkages can be circumvented by their replacement by an amide function. Furthermore, as a result of the presence of amine and acid moieties, SAAs are suitable for conventional combinatorial synthesis in both solid and solution phase. Many of these aspects have been extensively studied by Fleet’s group over the last 10 years. Efficient synthetic procedures for protected and unprotected homo-oligomeric derivatives of D-*arabino*- (Figure 5) [32–34] , D-*talo*- [35], L-*allo*- [35], D-*galacto*- [36], D-*allo*- [37], D-*lyxo*- [38], L-*xylo*- [39] and L-*ribo*-configured [39] tetrahydrofuran (THF) amino acids were optimised in order to study the influence of ring configuration and protecting groups on the secondary structure in these carbopeptoids.

**Figure 6:**
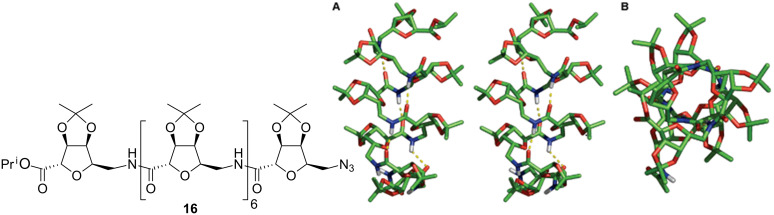
(A) Stereoview of the minimized structure of compound **16** (produced by a 500 ps simulation) that most satisfies the 27 NOE derived distance constraints. (B) View looking from *C* to *N* terminus. Hydrogen bonds are indicated by dotted lines.

Short oligomeric chains of *C*-glycosyl β-D-*arabino* THF amino acids **14** and **15** (where the C-2 and C-5 substituents of the THF ring are *cis* to each other) exhibit a well defined repeating turn secondary structure stabilised by inter-residual hydrogen bonds, whereas the epimeric α-D-*arabino*furanose oligomer **12** (with the C-2 and C-5 substituents in *trans* relative disposition) do not show any secondary structure in solution [34]. NMR and IR studies on D-*galacto*- [36] and D-*allo*-configured [37] oligomeric carbopeptoids demonstrated that the inversion of a single stereocentre on the THF ring can have more pronounced effects for solution conformations than the presence or absence of different protecting groups. Thus, 2,4-*cis*-THF-L-ribonate oligomers adopt hydrogen bond stabilised conformations whereas 2,4-*trans*-THF-L-xylonate oligomers do not. Additionally, a number of structurally related THF aminoacid oligomers were examined by chiroptical spectroscopy to aid interpretation of their conformational preferences. The use of CD, in addition to NMR and solution IR, enabled the classification of the conformations adopted by carbopeptoids as hydrogen bonded regular, non-hydrogen bonded regular and non-hydrogen bonded irregular [40]. “Regular” is used to define a conformation that is either hydrogen bonded or non-hydrogen bonded and not in equilibrium with other multiple conformations. If conformational exchange operates, the term “irregular” is used. For example, these studies demonstrated that an octameric chain of *C*-glycosyl α-D-*lyxo* furanose amino acids **16** adopts a regular hydrogen bonded conformation similar to an π-helix (Figure 6) [38], whereas α-D-*lyxo* [38], D-*talo* and L-*lyxo* tetramers have regular non-hydrogen bonded conformations [40]. Also, based on these techniques, Andreini et al. [41] have recently demonstrated that homo-oligomers of β-SAA (β-*N*-mannofuranosyl-3-ulosonic acid) adopt eight-membered ring hydrogen bonded double-turn regular conformations in solution.

Chakraborty’s group has also prepared protected and unprotected homo-oligomeric derivatives of D-*manno*- [42] and D-*gluco*-configured [43] tetrahydrofuran (THF) amino acids and studied their conformational properties by chiroptical and NMR techniques. Although both hydroxyl-protected linear oligomers displayed well-defined turn structures, their unprotected counterparts resulted in a less robust and regular conformation. However, in some cases the studies of the conformational preference in carbopeptoids is quite challenging and must be argued by computational methods [38,44] and infrared ion-dip spectroscopy [45] in order to interpret the data from CD, IR and NMR experiments reliably.

**Figure 7:**
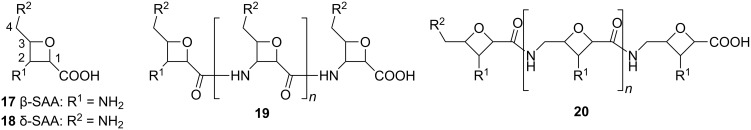
Structures of linear oxetane-β- and δ-SAA homo-oligomers **19**–**20**.

**Figure 8:**
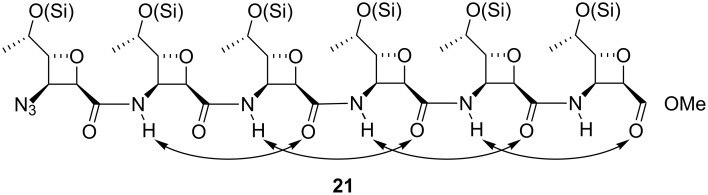
10-Membered ring H-bonds in compound **21** consistent with NMR and modelling investigations.

To complement its research programme on foldamer design and because of the absence of structural investigations on linear homo-oligomers constrained by 4-membered rings, Fleet and co-workers undertook the synthesis of carbopeptoids based on the oxetane template (Figure 7) [46–48]. Conformational analysis was carried out for two oxetane β-SAA hexamers with the 6-deoxy-L-*altro* and D-*arabino* configurations by means of detailed NMR and IR studies in combination with molecular mechanics [49]. These studies identified a left-handed helical secondary structure for the 6-deoxy-L-*altro*-oxetane hexamer and a right-handed helical structure for the D-*arabino*-configured oxetane hexamer **21** (having the opposite absolute configuration at C-2 and C-3) stabilised in both instances by 10-membered ring hydrogen bond arrangement (Figure 8).

Similar conformational studies combined with CD spectroscopy have been carried out for homo-oligomers of silyl-protected δ-2,4-*trans*-oxetane-SAAs with different configurations. The results showed that 6-deoxy-L-altronate, D-fuconate and 6-deoxy-D-gulonate oligomers are characterised by irregular non-hydrogen bonded conformations whereas L-rhamnoate and D-lyxonate oligomers have regular conformations governed by sterical interactions [50]. In contrast to the δ-2,4-*trans*-oxetane-SAA oligomers, the δ-2,4-*cis*-oxetane-L-ribonate tetramer and hexamer adopted a repeating β-turn structure [51] dictated by internal 10-membered hydrogen bonded rings. Such conformations are similar to those reported for the δ-2,5-*cis*-oxetane-THF oligomers [37].

**Figure 9:**
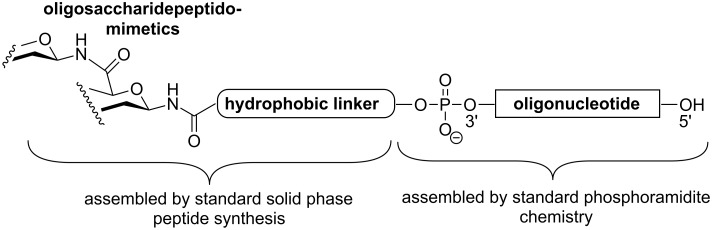
General structure of carbopeptoid-oligonucleotide conjugates.

D’Onofrio et al. [52] have carried out solid phase synthesis of oligonucleotides conjugated at the 3′ terminus with (1→6)-amide-linked oligosaccharide mimics (Figure 9). The presence of the saccharide unit at the 3′-end of 18-mers significantly enhanced the stability of the oligonucleotides in bovine fetal serum, non-negatively interfering with their ability to form stable duplexes with complementary DNA strands, as evaluated by UV thermal denaturation studies.

**Figure 10:**
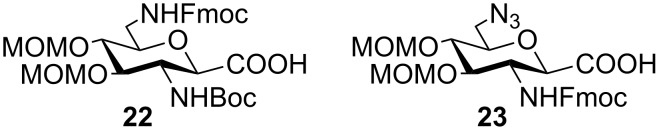
Protected derivatives of 2,6-diamino-2,6-dideoxy-β-D-glucopyranosyl carboxylic acid **22** and **23**.

Orthogonally protected sugar diamino acids (SDA) were first synthesised by Sicherl and Wittmann [53] and used to form linear and branched amide-linked mimetics (Figure 10).

Due to the various possibilities with which SDAs can be connected to each other, a high degree of diversity can be achieved by employing only a small set of different SDAs. Moreover, oligomeric SDAs with unprotected groups represent a novel type of aminoglycoside mimetics with potential recognition properties towards new RNA targets emerging in the post-genome era.

### Cyclic SAA homo-oligomers

**Figure 11:**
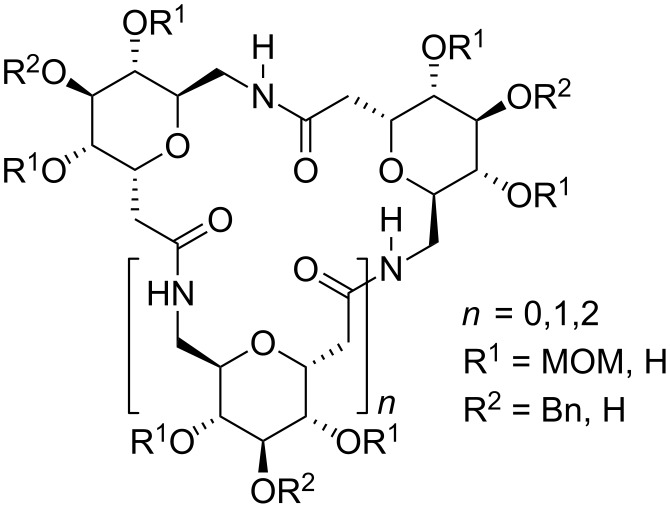
Cyclic homo-oligomers containing glucopyranoid-SAAs.

A cyclic array of desired ring size and defined secondary structure of alternating carbohydrate moieties and amide groups might conceivably lead to exquisite specificity of recognition and catalysis. One application of this concept is the mimicry of cyclodextrin inclusion complexes. Thus, Kessler and co-workers have reported the synthesis of cyclic oligomers containing glucopyranosyluronic acid by exploiting standard solid and solution phase coupling procedures [54]. These cyclic homo-oligomers of SAAs behave as host molecules that form inclusion complexes with *p*-nitrophenol and benzoic acid. Following the same objective, Xie’s group prepared orthogonally protected cyclic homo-oligomers with two to four SAA units that can be selectively or fully deprotected to afford macrocycles that can undergo further functionalisation (Figure 11) [55].

**Scheme 2:**
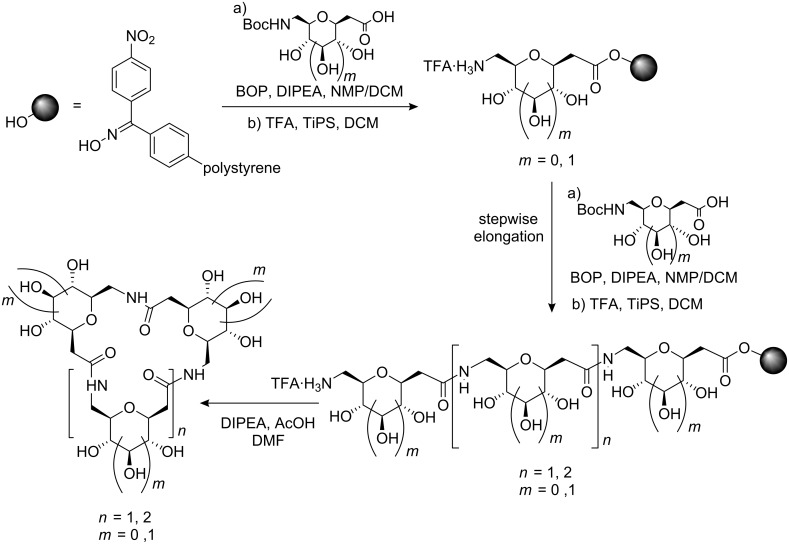
Strategy for solid-phase synthesis of cyclic trimers and tetramers containing pyranoid δ-SAAs.

Conformational analysis by molecular modelling showed that the unprotected cyclic trimers and tetramers preferred a ^4^*C*_1_ chair conformation with the oxygen atoms of the sugar ring located in the interior of the cavity and the secondary hydroxyl groups outside. This conformation is in agreement with the capacity of these cyclic carbopeptoids to form inclusion complexes with aromatic guest molecules [54] and Cu(II) ion [56]. Additionally, van Well et al. have described a cyclization/cleavage strategy for solid phase synthesis of cyclic trimers and tetramers containing pyranoid δ-SAAs (Scheme 2) and reported their structural analysis from ROESY data in combination with molecular dynamics calculations [57]. The results showed that the furanoid and the pyranoid SAA trimers adopt well-defined structures, although the trimer composed of pyranoid SAAs is less flexible than its furanoid counterpart.

Chakraborty and co-workers have also synthesised cyclic homo-oligomers of mannose- and glucose-derived furanoid SAAs [58] in order to constrain their conformational degrees of freedom and to induce desirable structural elements essential for their biological activities, such as tubular structures for transporting ions or molecules across membranes. Detailed ROESY, temperature coefficient (Δδ*/*Δ*T*) measurements of amide protons and constrained MD simulations revealed that all the cyclic oligomers had symmetrical structures, although none of the unprotected cyclic oligomers displayed any ability to transport ions across model membranes according to ion flux studies.

**Figure 12:**
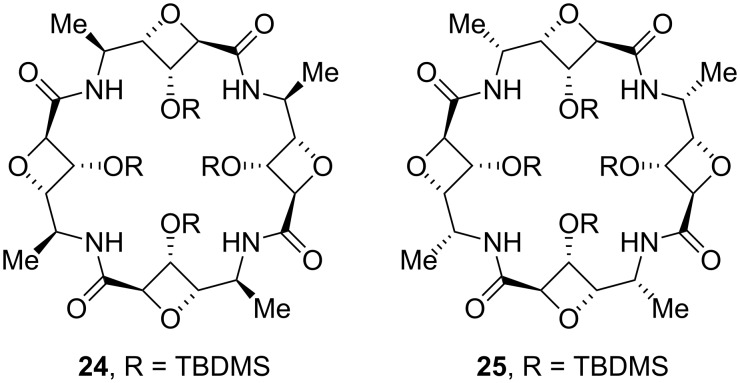
Cyclic tetramers of L-*rhamno*- and D-*gulo*-configured oxetane-SAAs.

Cyclic tetramers of L-*rhamno*- and D-*gulo*-configured oxetane-SAAs **24** and **25** (Figure 12) have been synthesised by Fleet et al. [59] as mimics of naturally occurring cyclic peptides and cyclodextrins, although no further conformational and recognition studies on these compounds have currently been published.

### Urea, carbamate and thiourea-linked sugars

The replacement of amide groups by pseudoamide (NH–C = X)–Y; X, Y = O, N, S) has been widely used in peptide chemistry to induce well-defined secondary structures. In carbohydrate chemistry, the incorporation of pseudoamide-type intersaccharide linkages has an additional interest because these functional groups are isosteric with phosphodiesther groups occurring in oligoglycosylphosphates and nucleotides.

**Figure 13:**
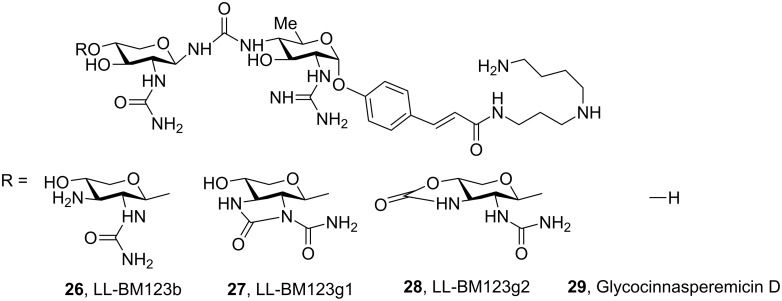
Aminoglycosidic antibiotics of the glycocinnamoylspermidine family.

Some examples of natural compounds having saccharide units connected by pseudoamide linkage are known. For example, the family of glycocinnamoylspermidine antibiotics **26**–**28**, whose structures were determined by Ellestad and co-workers [60] by NMR spectroscopy and X-ray diffraction, are characterised by the presence of a glycosylurea linkage (Figure 13). In 2005, Ichikawa and co-workers described the total synthesis of the glycocinnasperemicin D **29**, a broad-spectrum antibiotic against Gram-negative organisms, which contains two highly functionalised aminosugars connected by an urea linkage [61]. While exploring the synthesis of this target molecule, this group established a new method for the stereoselective synthesis of novel β-urea-linked pseudooligosaccharides, which involves the reaction of amine-glycosides with Steyemark-type gluco- and galactopyranosyl oxazolidinones [62–63].

**Scheme 3:**
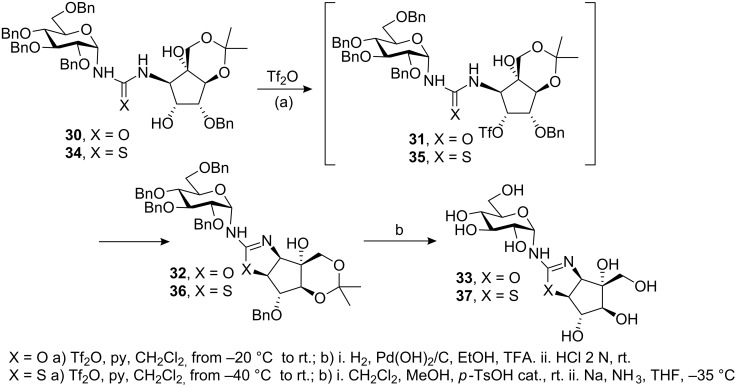
Synthesis of (thio)trehazoline, via triflate, from β-hydroxy(thio)urea.

Another example of natural disaccharide analogue containing a pseudoamide linkage is trehazolin **33** [64], which possesses a cyclic isourea functionality between the α-D-glucose and aminocyclopentitol rings. Trehazolin is a potent trehalase inhibitor in which an aminocyclopentitol ring replaces the glucopyranosyl cation postulated as an intermediate in the enzymatic hydrolysis of α,α-trehalose. Although several synthetic methodologies had successfully been applied to prepare this inhibitor, Chiara and co-workers have described a novel complementary approach in which the oxazoline ring is generated by S_N_2 nucleophilic displacement reaction from the β-hydroxyurea **30** via the triflate intermediate **31** (Scheme 3) [65].

A similar methodology was used by the same authors to prepare the trehazolin analogue **37** with an isothiourea group instead of an isourea group, which is also a potent trehalase inhibitor [66–67]. D-*Gluco* trehazolin analogues have also been synthesised by means of novel methodology based on the coupling of *O*-unprotected β-D-glucopyranosyl isothiocyanate with different aminosugars followed by treatment with yellow mercury (II) oxide [68].

**Figure 14:**
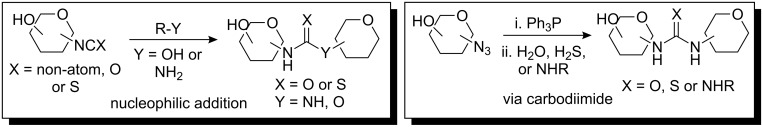
Approaches to access pseudoamide-type oligosaccharide mimics.

There are two general strategies to access pseudoamide-type oligosaccharide mimics: i) nucleophilic addition of sugar derivatives to carbohydrate isocyanates, isothiocyanates or isocyanides; and ii) conversion of sugar azides into glycosyl carbodiimides via a tandem Staudinger-aza-Wittig type reaction with triphenylphosphine and an isothiocyanate, followed by the addition of a nucleophile (H_2_O, H_2_S, NH_3_, amines) (Figure 14). Although this second methodology is more straightforward and versatile than the first, only a few examples of pseudooligosaccharides having cyanamide, urea and thiourea-linkages via the Staudinger reaction have been reported [69–70].

**Figure 15:**
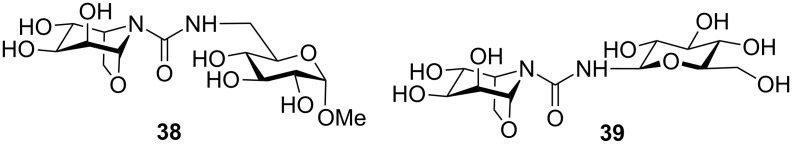
Calystegine B_2_ analogues **38** and **39** with urea-linked disaccharide structure.

In our group, the carbodiimide approach was used to prepare calystegine B_2_ analogues with the urea-linked disaccharide structure (Figure 15). These compounds, however, did not show inhibitory activities, probably due to the presence of a hydrophilic sugar substituent at the nitrogen atom of the *nor*-tropane ring [71–72].

Several groups have shown that disaccharide ureas and carbamates with different bridging positions as α/β-anomers are readily accessible by the coupling reaction of aminosugars or sugars, respectively, and glycosyl-isocyanates [73–74] or isocyanides [75]. However, the experimental difficulties in handling isocyanates has led to the preferential use of sugar carbodiimides as key intermediates for the preparation of glycosylureido sugars [76–79].

**Figure 16:**
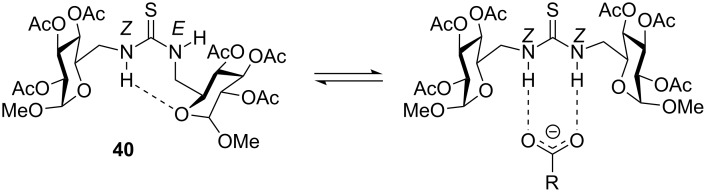
Rotameric equilibrium shift of **40** by formation of a bidentate hydrogen bond.

From the large number of functional groups that can be employed as surrogates for the natural amide and phosphodiester linkages, the thiourea functionality ranks among the most popular. Firstly, it can generally be generated in high yield and are ideally suited for solid phase and combinatorial approaches. Secondly, thioureas can be easily transformed into carbodiimides and thus serve as key intermediate in the synthesis of other pseudoamide functionalities (urea, guanidine, etc.) [70–72,76–79]. Thirdly, thiourea bridges provide efficient anchoring points for bidentate hydrogen-bonding recognition, which can give rise to defined secondary structures or be exploited in molecular recognition. Conformational studies by temperature coefficient measurements and ROESY experiments showed that thioureidodisaccharides adopt the *Z,E* conformation at N–(C=S) bonds, which is stabilised by intramolecular hydrogen bonding with the formation of a 7-membered ring (e.g. **40**) [80]. This conformation changes to the *Z,Z*-conformation in aqueous solution [70] or in the presence of carboxylate ligands due to the formation of a bidentate hydrogen bond (Figure 16) [80].

**Figure 17:**
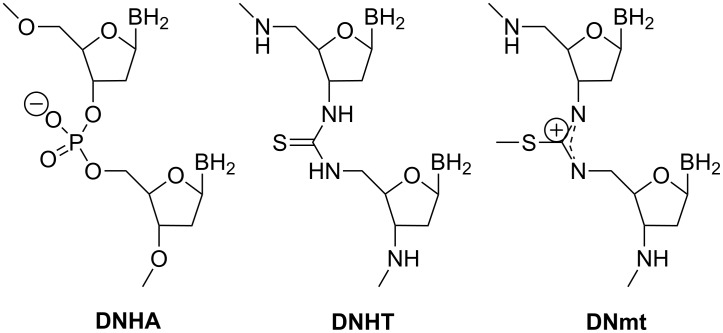
Nucleotide analogues with thiourea and *S*-methylisothiouronium linkers.

Examples of thiourea-connected saccharides have been typically limited to compounds where the tethering reaction involves only two moieties, with no possibility of chain elongation [68,70–72,81], except in cases where there are spacer substituents [82]. Interestingly, a thiourea-linked dinucleotide was elongated by solid-phase synthesis to incorporate positively charged isothiouronium inter-nucleoside linkages into otherwise negatively charged DNA (Figure 17) [83–84]. The binding of these artificial DNAs (DNmt or DNT) to its complementary DNA strand occurs as with the unmodified DNA·DNA duplex but with enhanced nuclease resistance.

**Scheme 4:**
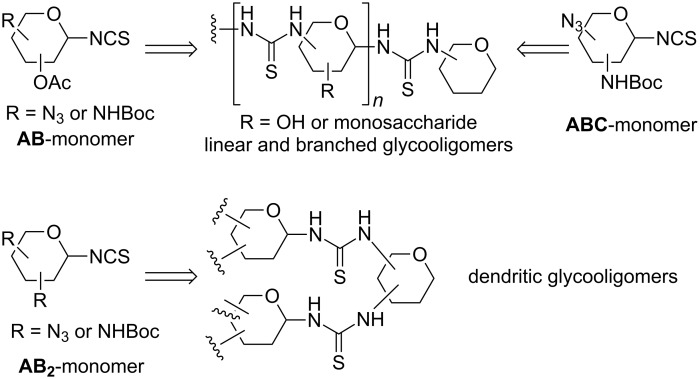
Retrosynthetic approach to synthesize thiourea-linked glycooligomers.

**Figure 18:**
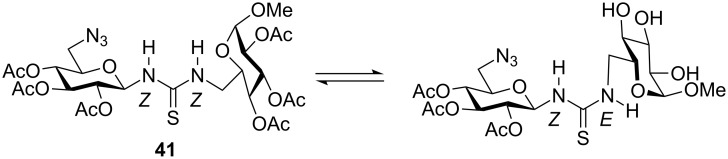
Rotameric equilibria for β-(1→6)-thiourea-linked glucodimer **41**.

In order to design longer thiourea-linked glycooligomers with linear dendritic and branched architectures, our group has recently reported an efficient synthetic strategy based on the use of **AB**, **AB_2_** and **ABC**-type monosaccharide building blocks containing isothiocyanate (**A**), azido (**B**) or carbamate groups (**C**) (Scheme 4) [85,87]. An iterative and efficient three-step reaction sequence was described for the assembly of monosaccharide units that involves: (i) a thiourea-forming reaction; (ii) deprotection of the hydroxy groups in the adducts and (iii) the generation of a new amino group in the growing chain. Branching points are incorporated by inserting building blocks bearing orthogonal amine functionalities at specific locations in the chain [86]. Conformational studies by dynamic NMR spectroscopy of β-(1→3)- and β-(1→6)-linked diglucosylthioureas (e.g. **41**) detected the presence of *Z,Z* and *Z,E* conformers at the N–(C=S) bond (Figure 18).

β-(1→6)-Linked thioureido-di- and trisaccharides were used to obtain the corresponding ureido- and guanidine-linked oligomers. These compounds were evaluated as phosphate binders in water [76]. Association constants (*K*_as_) for the binding of dimethyl and, especially, phenyl phosphate were obtained from NMR titration experiments for both series of glucooligomers. The binding affinity of the thiourea and guanidine oligomers was stronger than in the case of the urea analogues.

**Figure 19:**
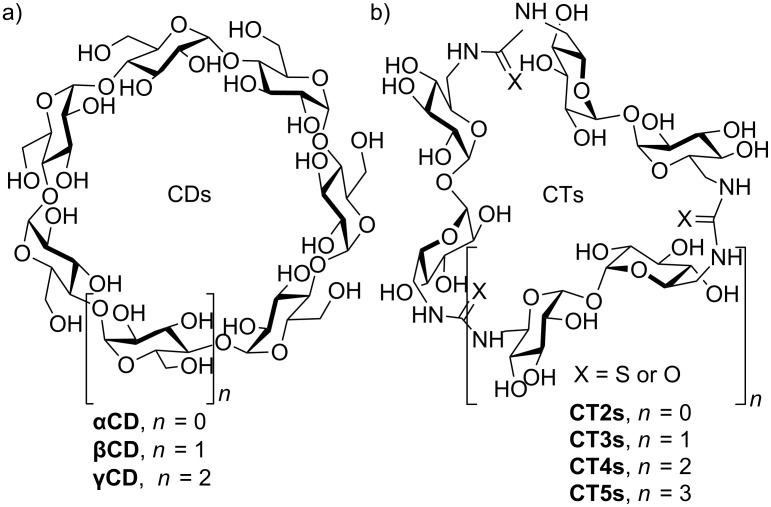
Schematic representation of (a) cyclodextrin (CDs) and (b) cyclotrehalan (CTs) family members.

Stimulated by the interesting supramolecular properties and applications of cyclodextrins (CDs), a range of novel host molecules with differently shaped internal cavities has been obtained by replacement of the natural glycosidic bonds by urea and thiourea linkages. Thus, our group developed an efficient and modular strategy for the synthesis of cyclopseudooligosaccharide receptors relying on alternating α,α-trehalose motifs and semi-rigid thiourea segments (cyclotrehalans, CTs) [77–79,88–89]. Molecular diversity was introduced at the inter-saccharide connectors by exploiting the chemistry of macrocyclic carbodiimides [77–79,88–89] as well as by varying the size of the macrocycle (Figure 19).

Molecular modelling predicts the existence of a flexible, relatively hydrophobic cavity suited for molecular inclusion of non-polar guests [79,88]. NMR supports this fact and confirms the high symmetry of these hosts, where all glucopyranosyl units are magnetically equivalent as in the case of cyclodextrins. Determination of the inclusion capabilities towards a series of structurally diverse guests demonstrated that large-ring CTs are well suited for forming supramolecular complexes in water [77,79,88].

### Guanidine-linked sugars

The use of the guanidine functional group to connect monosaccharide units in glycooligomers is particularly attractive with regard to molecular recognition processes. Like thioureas and ureas, guanidines can also form bidentate hydrogen bonds. In addition, because of their positively charged character, guanidines can exert strong electrostatic interactions with negatively charged functional groups such as phosphate or carboxylate groups in DNA, RNA and proteins.

**Scheme 5:**
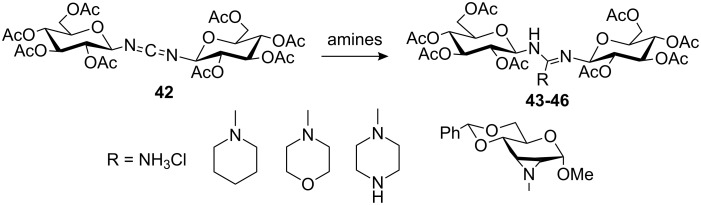
Synthesis of guanidine-linked pseudodisaccharides via carbodiimide.

Tóth and co-workers [90] synthesised a series of guanidine-linked sugars as a new class of oligosaccharide mimics to study their interactions with proteins. Thus, reaction of the carbodiimide-linked pseudooligosaccharide **42** with different secondary amines gave the corresponding guanidines **43**–**46** (Scheme 5) and their conformational behaviour and tautomerism was studied by NMR spectroscopy.

**Figure 20:**
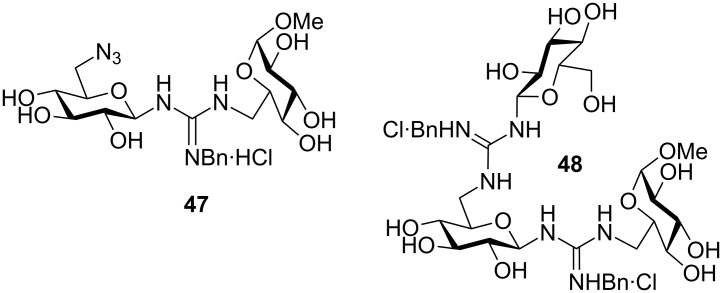
β(1→6)-Guanidine-linked pseudodi- and pseudotrisaccharides **47** and **48**.

β(1→6)-Linked pseudodi- (**47**) and pseudotrisaccharides (**48**) incorporating guanidine intersaccharide bridges have been prepared and evaluated as phosphate binders in water (Figure 20) [76]. Association constants (*K*_as_) for binding with dimethyl and phenyl phosphate were obtained from NMR titration experiments for both compounds. The results indicated that the binding strength depends strongly on the acidity of the NH protons and on solvating properties. The guanidine derivatives showed higher *K*_as_ values than the isosteric thiourea and urea analogues.

**Scheme 6:**
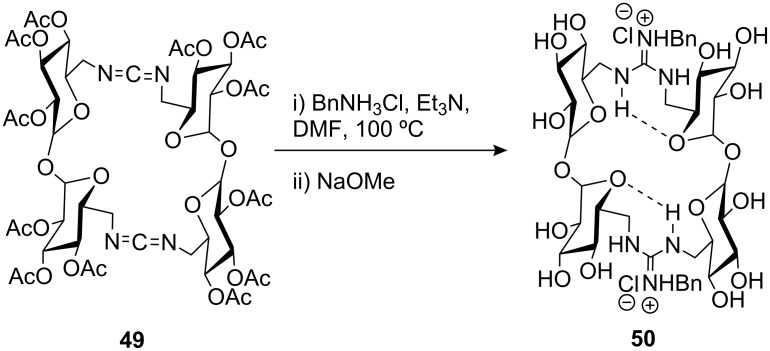
Synthesis of *N*-benzylguanidine-linked CT2 **50**.

Ortiz Mellet’s group has reported the synthesis of *N*-benzylguanidine-linked dimeric cyclotrehalans (CT2s, **50**) [78] via carbodiimide **49** by reaction with benzylamine hydrochloride and subsequent deacetylation (Scheme 6). Structural and conformational studies on **50** were carried out by NMR, which showed that the structure was stabilised by two anti-parallel seven-membered ring intramolecular hydrogen bonds, resulting in relatively high rotational barriers for the *Z,E:E,Z/E,Z:Z,E* equilibrium. The cavity collapses due to the presence of these intramolecular hydrogen bonds thus preventing the formation of inclusion complexes.

**Figure 21:**
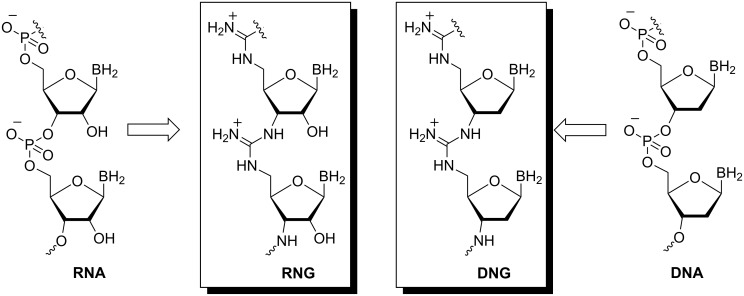
Structure of **RNG** and **DNG**.

Most examples of linear pseudoamide-linked glycooligomers correspond to oligonucleotide analogues. The “antisense” strategy to regulate gene expression in living cells has required the development of modified oligonucleotides as potential therapeutic agents. A key goal in the design of such agents include increasing binding affinity while maintaining sequence specificity, resistance to degradation by nucleases and improved membrane permeability [83–84]. Numerous structural analogues of DNA/RNA designed to be effective antisense/antigene agents have been reported. An interesting approach involves replacing the negatively charged phosphodiester linkages of RNA/DNA by positively charged guanidinium linkages. The guanidinium linkage is resistant to nucleases [91–92] and the positive charge may give rise to cell membrane permeability through electrostatic attraction with the negatively charged groups of the proteoglycans at the cell surface. Using this strategy, Bruice and co-workers synthesised ribonucleic guanidine (RNG) and deoxyribonucleic guanidine (DNG) as analogues of RNA and DNA, respectively (Figure 21).

**Figure 22:**
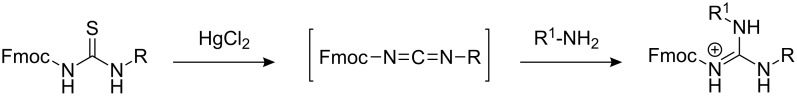
Preparation of Fmoc-guanidinium derivatives.

The methodology developed to generate internucleoside guanidinium linkages can be used in both solution phase and solid phase synthesis [91–94]. It involves the abstraction of the sulphur atom from a (Fmoc)-protected thiourea by Hg^2+^ to provide an activated carbodiimide which can react with a free amino group to give the protected guanidine (Figure 22).

**Figure 23:**
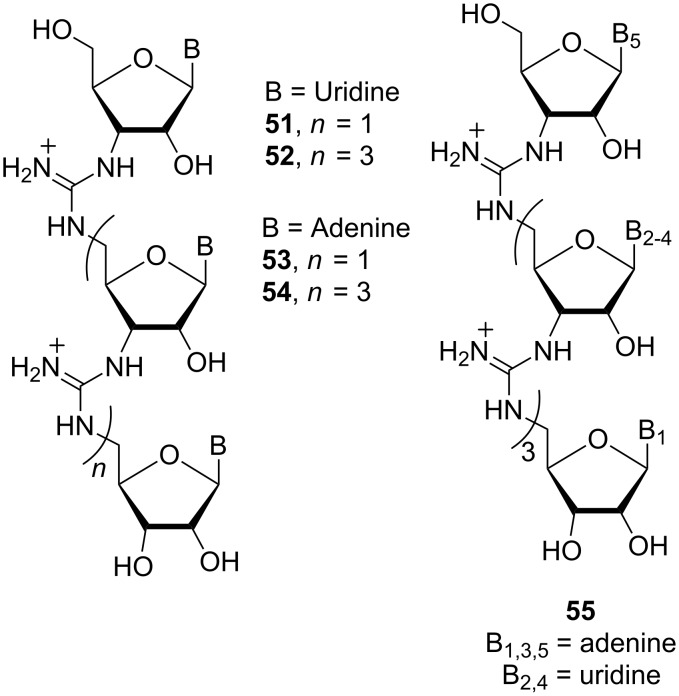
Structures of the homo-oligomeric RNG derivatives **51**–**55**.

**Figure 24:**
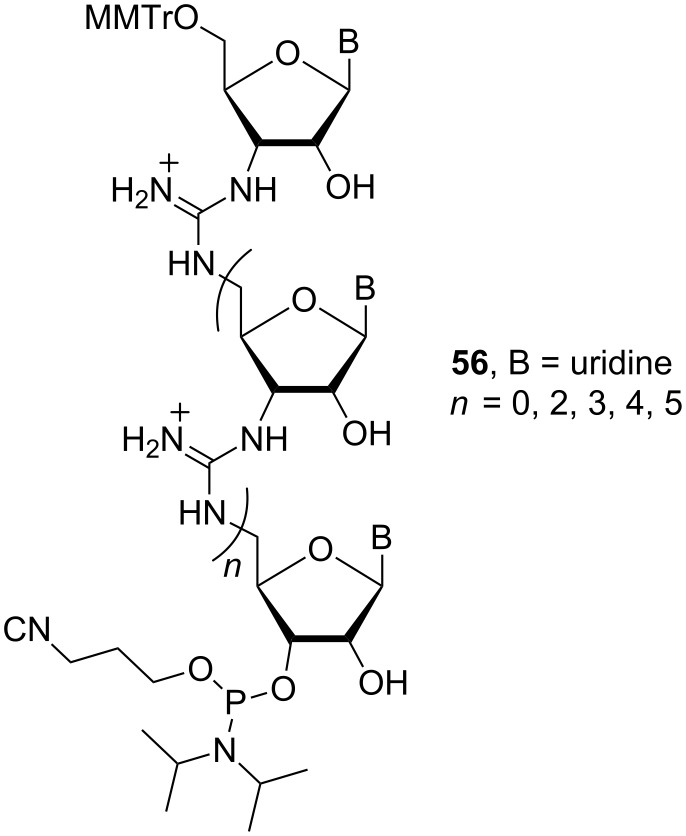
Phosphoramidite building block **56**.

Homo-oligomeric RNG sequences, containing trimeric and pentameric uridyl [95] and adenyl moieties (**52**-**55**) (Figure 23), and a mixed-base pentamer [96] (**56**, 5′-AUAUA-3′), were synthesised using this methodology (Figure 24).

Binding studies of uridyl ribonucleic guanidine **52** to DNA [97] and RNA [98] showed that it binds pentameric adenyl DNA and RNA with a 1:1 stoichiometry. The RNG **52** dimeric complex with DNA is thermodynamically favoured in comparison to RNA·DNA and DNA·DNA duplexes and was able to discriminate between complementary and non-complementary base pairs. These results suggest that **52** is a good candidate for an antisense agent.

A 21 base pair RNG/DNA chimera containing both anionic phosphodiester linkages of DNA and cationic guanidine linkages of RNG has been synthesised [99]. Phosphoramidite **56** (Figure 24) was used as a building block to introduce guanidinium linkages at desired positions in the chimeric oligonucleotides. The biological properties were evaluated using the bcr-abl oncogene (the cause of chronic myeloid leukaemia) as the target. The results showed that the binding of a 21-mer RNG/DNA chimera containing six guanidinium linkages is more than 104-fold stronger than the binding of its 21-mer DNA counterpart.

**Figure 25:**
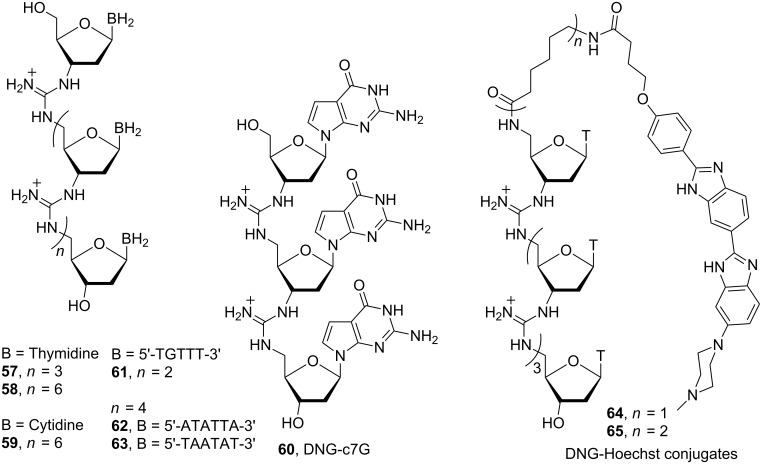
Structures of DNGs **57**–**65**.

Deoxyribonucleic guanidine homo-oligomeric sequences, consisting of trimeric and octameric thymidyl [100], octameric cytidyl [101] and trimeric 7-deazaguanidyl moieties (**57**–**60**) [102] and a mixed-base tetramer [103] and hexamer [104] (**61**–**63**) were synthesised using the same strategy as noted above for RNG synthesis (Figure 25).

Binding studies of homo-oligomers **57** and **58** to complementary DNA oligomers showed a 1:2 DNG:DNA stoichiometry, similar to homo-polymeric thymidyl and adenyl DNA strands, which can form a triple helical structure. On the other hand, octameric cytidyl DNG **59** and mixed-based oligomers **61**–**63** form 1:1 complexes with complementary DNA oligomers. The trimeric 7-deazaguanidyl **60**, in which guanine is replaced by 7-deazaguanine (c^7^G), forms a 1:1 complex with pentameric adenyl DNA. Deazaguanine nucleobase was chosen because of the unique glycoside bond stability and its ability to prevent G-quartet formation.

To increase the already strong binding of DNG to DNA, DNG was combined with a ligand capable of binding specifically to the minor groove of the DNA. A pentameric thymidyl DNG incorporating bis-benzimidazole (Hoechst 33258) ligand (**64**) was synthesised [105]. The stability of DNG-Hoechst conjugates **64** and **65** with a 30-mer double-strand DNA (dsDNA) and single-strand DNA (ssDNA) were evaluated. Fluorescent emission studies showed that hybridization of DNG-Hoechst conjugates **64** and **65** to dsDNA enhances the stability of the triple helix through simultaneous minor groove binding by the tethered Hoechst 33258 ligand. Furthermore, Hoechst 33258 is able to enhance the stability of the duplex DNG·DNA with a flexible minor groove. As in the case of RNG, a 20 base pair DNG/DNA chimera has been synthesised [106].

**Figure 26:**
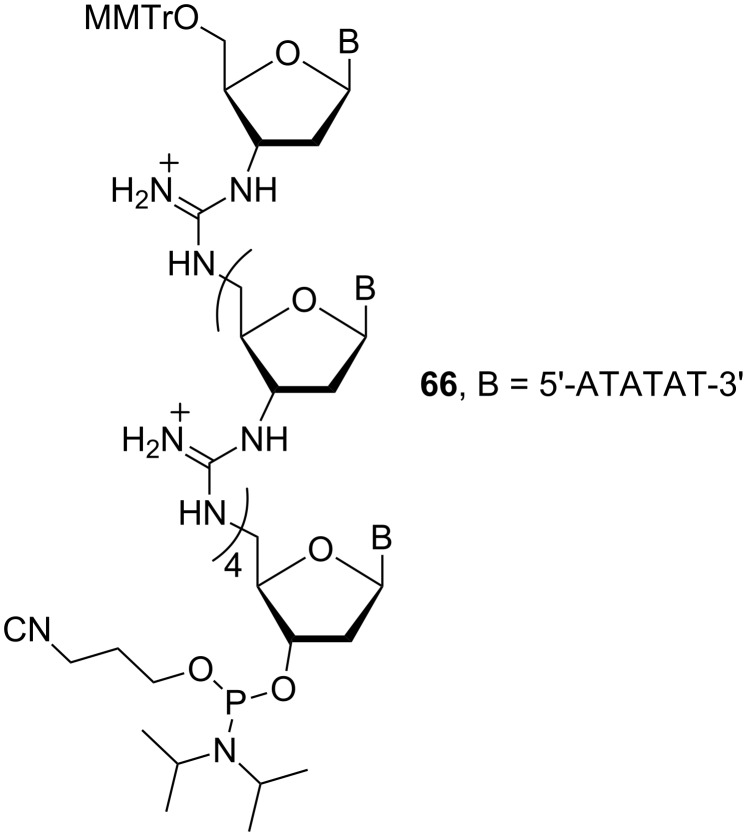
Structure of the phosphoramidite building block **66**.

Phosphoramidite **66** (Figure 26) was used as the starting material for the introduction of guanidinium linkages at desired positions in the chimeric oligonucleotides. The biological properties were evaluated using the juvenile esterase gene as the target. The results showed that binding of a 20-mer DNG/DNA hexameric (ATATAT) chimera containing guanidinium linkages is more than 10^5.7^ times stronger than the binding of the corresponding 20-mer of DNA. The hexameric DNG binds to DNA in a 1:1 ratio and is able to discriminate between complementary and non-complementary base pairs. In addition, the DNG·DNA complex is more stable than the DNA·DNA duplex.

Cancer cell immortality is due to relatively high concentrations of telomerase enzyme which maintains the telomer sequence during cell division. One approach to prevent telomer replacement in cancer cells is by inhibiting the telomerase enzyme by blocking the telomeric RNA 11-base template (5′-CUAACCCAAC-3′). For this purpose, Bruice and co-workers [107] prepared complementary DNG (5′-GATTGGGATTG-3′) to telomeric RNA complex. Binding studies demonstrated that the DNG/telomeric RNA complex is favoured over the telomer substrate, and could be of use as an anticancer agent.

## Conclusion

Over the last 10 years considerable efforts have been expended in the synthesis of novel linear and cyclic pseudoamide-linked oligosaccharide mimics. In particular, important advances in synthetic methodologies for thioureido and ureido glycooligomers have made thus enabling the preparation of virtually any oligomeric structure (linear, branched, dendritic and cyclic). The secondary structures adopted by these pseudooligosaccharides have been extensively studied by current NMR and molecular modelling techniques. The results have shown that, in many cases, the three-dimensional structure of naturally occurring biomolecules can be mimicked by carbopeptoids and pseudoamide-linked oligomeric surrogates. Nevertheless, further investigations are required to clarify the structure-activity relationships in order to design novel biologically active analogues of potential therapeutic value.
